# The Neglected Marine Fungi, *Sensu stricto*, and Their Isolation for Natural Products’ Discovery

**DOI:** 10.3390/md17010042

**Published:** 2019-01-10

**Authors:** David P. Overy, Teppo Rämä, Rylee Oosterhuis, Allison K. Walker, Ka-Lai Pang

**Affiliations:** 1 Ottawa Research and Development Centre, Agriculture and AgriFood Canada, Ottawa, ON K1A 0C6, Canada; 2 Marbio, Norwegian College of Fishery Science, University of Tromsø—The Arctic University of Norway, 9019 Tromsø, Norway; teppo.rama@uit.no; 3 Department of Biology, Acadia University, Wolfville, NS B4P2R6, Canada; 117135o@acadiau.ca (R.O.); allison.walker@acadiau.ca (A.W.); 4 Institute of Marine Biology and Centre of Excellence for the Oceans, National Taiwan Ocean University, 20224 Keelung, Taiwan; klpang@mail.ntou.edu.tw

**Keywords:** marine fungi, isolation, culturing, identification, natural products, secondary metabolites

## Abstract

Despite the rapid development of molecular techniques relevant for natural product research, culture isolates remain the primary source from which natural products chemists discover and obtain new molecules from microbial sources. Techniques for obtaining and identifying microbial isolates (such as filamentous fungi) are thus of crucial importance for a successful natural products’ discovery program. This review is presented as a “best-practices guide” to the collection and isolation of marine fungi for natural products research. Many of these practices are proven techniques used by mycologists for the isolation of a broad diversity of fungi, while others, such as the construction of marine baiting stations and the collection and processing of sea foam using dilution to extinction plating techniques, are methodological adaptations for specialized use in marine/aquatic environments. To this day, marine fungi, *Sensu stricto*, remain one of the few underexplored resources of natural products. Cultivability is one of the main limitations hindering the discovery of natural products from marine fungi. Through encouraged collaboration with marine mycologists and the sharing of historically proven mycological practices for the isolation of marine fungi, our goal is to provide natural products chemists with the necessary tools to explore this resource in-depth and discover new and potentially novel natural products.

## 1. Introduction

The field of natural product discovery is under transition as genome sequencing and different ‘omics’ techniques gain more prominence within the research field. Techniques such as transcriptomics and metabolomics, which are used to reveal expressed genes and associated metabolites, are now being applied as tools in the discovery and expression of natural products. ‘Omics’-based natural product discovery pipelines are still in their infancy, but have shown their potential in finding new bioactive molecules without culturing the producing organism [1]. However, the isolation and culturing of filamentous fungi and other microorganisms will continue to have relevance in the field for many more years to come and, for many applications, will remain a necessity. For this reason, current and future generations of natural product chemists should know how to isolate, maintain, and preserve fungal cultures.

Filamentous fungi are a proven source of structurally diverse natural products. Few opportunities to explore truly virgin lineages of filamentous fungi for natural products remain; marine fungi in the strict sense (*Sensu stricto, s.s.*) comprise one of these still relatively underexplored areas. Marine fungi *s.s.* are those fungi that are exclusively found to occur in the marine environment, many of which have evolved specialized adaptations that aid in spore dispersal in marine waters. Reports of chemistry from marine fungi *s.s.* suggest that they are an excellent source of new chemical entities, often associated with a variety of different biological activities [2]. For this reason, over the past few decades, many research groups have focused their attention on the marine environment as a source of fungi for natural products discovery. As a result, an exponential increase in the publication of natural products from “marine-derived” fungi has occurred [3,4]. Unfortunately, the majority of most frequently cited marine-derived taxa belong to well-known osmotolerant/halotolerant generalist genera associated with ruderal substratum relationships, such as *Aspergillus* and *Penicillium*, while the discovery rate of new natural products from acknowledged marine fungi *s.s.* has not increased since the late 1990s [2]. Why the disparity?

Cultivability remains one of the main limitations associated with the discovery of natural products from marine fungi *s.s.* [5]. Isolation techniques commonly reported in the literature by marine natural products groups are dominated by simple plating methods, approaches that favor the isolation of faster growing generalist, osmotolerant/halotolerant fungal genera. These methods are antiquated and do not differ significantly from those practiced in the 1940’s and 50’s [2,6]. The implementation of focused strategies aimed at the isolation of marine fungi *s.s.* are required to tap into this underexplored resource and address the goal of discovering new bioactive natural products. The majority of natural products isolated from marine fungi *s.s.* have resulted from collaborations between natural products chemists and mycologists, where specialized strategies for the isolation of marine fungi *s.s.* were applied. The motivation of this review is to present a guide of best practices for the marine natural products community to implement and increase the discovery rate of natural products from exceptional marine fungi *s.s.* (summarized in Table 1).

## 2. Sources of Marine Fungi

**Figure 1:**
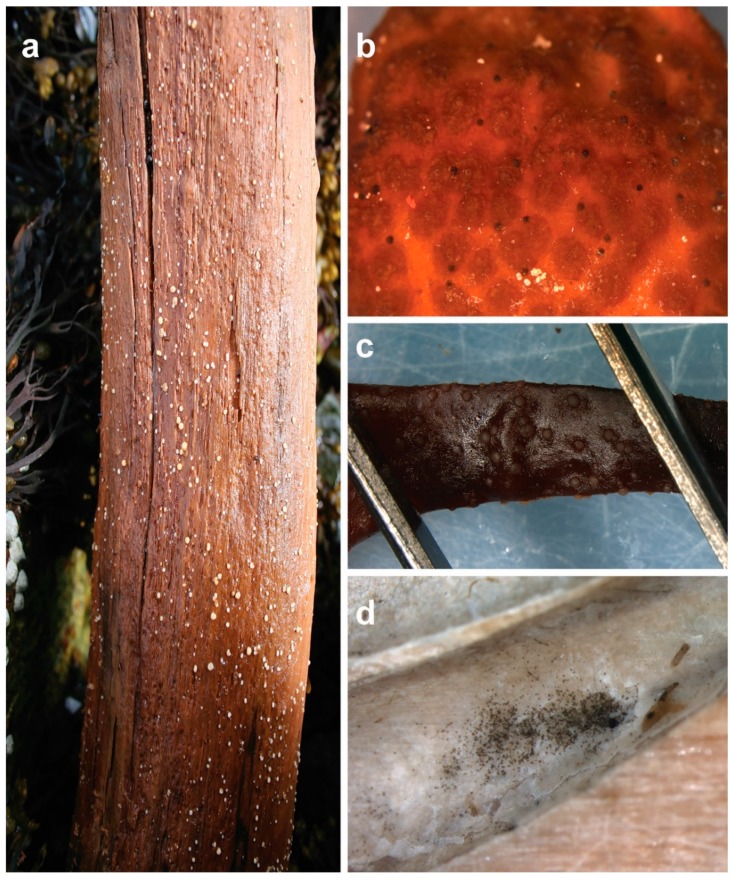
Fruiting structures of marine fungi: (**a**) *Amylocarpus encephaloides* is one of the few species of marine fungi that can be spotted and identified with the naked eye already in the field; (**b**) these “black dots” on the receptacle of macroalga *Ascophyllum nodosum* are fruiting bodies of *Stigmidium ascophylli* (syn. *Mycosphaerella*/*Mycophycias ascophylli*); (**c**) apothecial fruiting bodies of *Calycina marina* on dead and decaying *A. nodosum*; (**d**) conidial mass of the asexual form of the fungus, *Lulwoana uniseptata* (=*Zalerion maritima*), in a borehole made by a *Xylophaga* bivalve species.

Filamentous fungi are major decomposers of woody and herbaceous substrata entering marine ecosystems and propagate by spores, either asexual conidia or by sexual ascospores, basidiospores, or zygospores. In marine ecosystems, spores are dispersed and passively transported through water, ultimately landing on a suitable substrate where they germinate and colonize. During colonization, hyphae (fungal filaments) grow throughout the substrate and, when environmental conditions are appropriate, differentiate into specialized structures to reproduce. Observations of fruiting structures, such as pycnidia or ascomata (typically observed as round “balls” or “tiny dots” emerging from or forming on the substrate surface) and conidiophores (“branch-like” structures bearing wet or dry masses of conidia), are sought after by mycologists for isolating and identifying marine fungi (Figure 1). 

**Figure 2:**
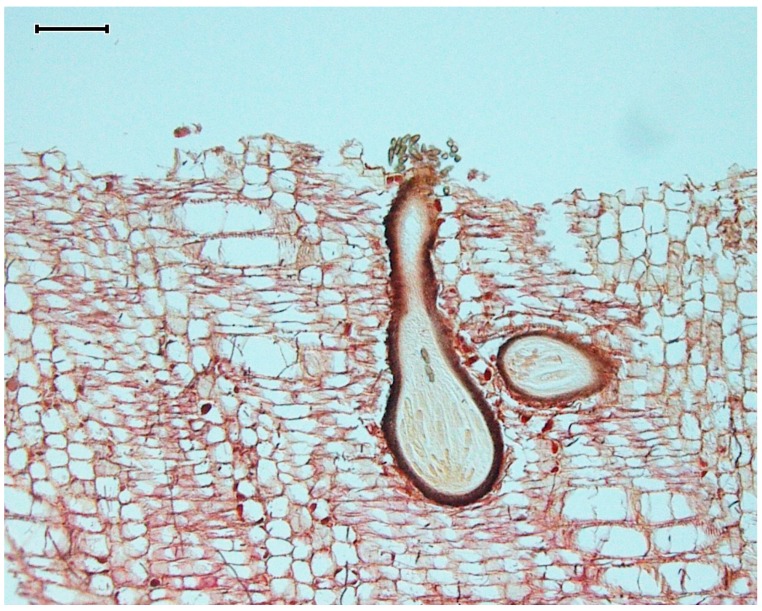
A cross-section micrograph of driftwood-embedded *Dyfrolomyces marinospora* ascomata (fruiting bodies) releasing ascospores (scale bar = 100 µm).

Marine fungi are found on a diverse range of substrates: Submerged wood, estuarine plants (such as mangroves and salt marsh grasses and rushes), sand grains, sediment, algae, animals (invertebrates and vertebrates), plankton, and probably even the plastisphere [7,8]. Unlike “marine-derived fungi”, many marine fungi *s.s.* have evolved specialized adaptive strategies to ensure successful spore dispersal in the marine environment. Ascomata of arenicolous marine fungi lack long central necks (that would be abraded by the constant movement of the sand grains) and associated ascospores are appendaged and often found to be trapped in sea foam [7,9]. Submerged wood substrata tend to favor the growth of members of the Halosphaeriaceae, Torpedosporales, and Lulworthiales (taxonomic groups in the fungal kingdom composed predominantly of marine fungi), especially in open oceans, where species are typically characterized by passive ascospore release (deliquescing asci) and appendaged ascospores that aid in floatation and attachment [10]. When occurring on submerged wood, ascomata are found partly or completely immersed within the substrata (Figure 2) [9]. Marine Ascomycota are adapted to life in the intertidal zone, and often release spores through the ostiole during periods of low tide, which rest upon the ascomata and become washed off into the water column during high tide, and their ascospores often have gelatinous sheaths (Dothideomycetes), appendages (Sordariomycetes), or, in some species, both to aid in the attachment of spores to substrata [7]. Many conidia (asexual spores) of marine fungi are branched or ornamented or extremely long in size to aid in dispersal and floatation, many of which can be found trapped in seafoam along the shoreline [11].

Marine fungi abound in intertidal zones, such as mangrove ecosystems, due to the associated and abundant organic detritus; over 280 species of fungi have been described from submerged mangrove substrata [7]. Algae dominate marine habitats in temperate regions and approximately 80 fungal species have been associated with algae either as parasites or symbionts [12]; however, algae as a source of marine fungal isolates have been grossly neglected and further isolation efforts will likely yield undiscovered species diversity [7]. Estuarine (or salt) marshes occur in the intertidal zone and are dominated by the growth of salt-tolerant plants. Few species of salt-tolerant plants have been surveyed; *Juncus roemerianus* and *Spartina* species are an exception (due to their dominance in this ecosystem) and are associated with a rich diversity of fungal species [13,14,15,16,17].

Although marine fungi in temperate and tropical areas are the most studied, a rich and diverse mycota exist in polar areas [18,19,20,21,22]. Fungi in these cold environments are associated with similar substrate relationships as in warmer regions. Sea-ice, however, is a habitat unique to polar marine regions and supports a diverse community of microorganisms, including fungi. Members of Chytridiomycota and Dikarya (Ascomycota and Basidiomycota) can be found dwelling in high abundance in sea-ice [18,19,20]. Studies targeting fungi in macroalgae in Antarctica have revealed high fungal richness [21]. In the Arctic, 100 species of marine fungi have been morphologically detected, whereas molecular data indicates the presence of thousands of fungi in the Arctic marine environment [22]. 

## 3. Natural Products from Marine Fungi (*Sensu stricto*)

**Figure 3:**
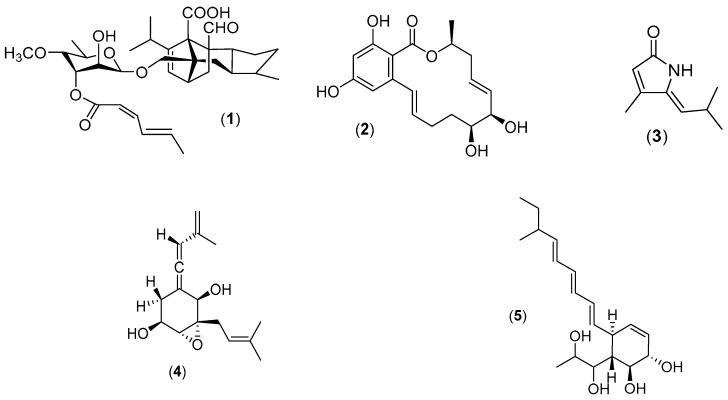
New natural products of marine fungi *s.s.*: zofimarin (**1**), aigialomycin D (**2**), pulchellalactam (**3**), spartinoxide (**4**) and spartinol C (**5**).

The phylogenetic breadth of marine fungi *s.s.* within the fungal kingdom is extensive, including marine-specific yeasts, chytrids, and other basal fungal groups; however, fungi in the Dikarya (ascomycetes and basidiomycetes) and the Zoopagomycotina are richer in the number and diversity of secondary metabolite gene clusters and therefore are those most likely to yield the greatest diversity of natural products [23,24,25]. Filamentous marine fungi (ascomycetes in particular) have proven to be a plentiful source of new natural products (for a detailed review refer to Overy et al., 2014) [2]. For example, from an isolate of *Aigialus parvus* (a marine ascomycete occurring on submerged wood), investigators obtained a total of 11 new natural products representing several different structural classes (aigialomycins A–G, aigialospirol and associated derivatives, and aigialone) [26,27,28,29,30,31]. *Halorosellinia oceanica* (syn. *Hypoxylon oceanicum*), a marine ascomycete associated with intertidal wood in mangrove habitats, studied by Wyeth for associated antifungal properties, led to the discovery of three new lipodepsipeptides (15G256ε, 15G256γ, 15G256δ), five new macrocyclic polyethers (15G256α, 15G256β, 15G256α-1, 15G256ι, and 15G256ω), and five new linear polyesters (15G256α-2, 15G256β-2, 15G256ο, 15G256ν, and 15G256π) [32]. New natural products obtained from marine fungi are associated with a range of biological activities (representative structures are presented in Figure 3). For example, an isolate of the obligate marine species, *Zopfiella marina,* isolated from marine mud (obtained from a depth of 200 m) was found to produce the potent antifungal metabolite, zofimarin, a sordarin derivative [33,34]. Aigialomycin D demonstrated antiplasmodial activity and cytotoxicity against human cell lines (where cytotoxicity has been attributed to cyclin-dependent kinase/glycogen synthase kinase-3 inhibition) [26,35]. The metabolite, pulchellalactam, obtained from a *Corollospora pulchella* strain isolated from driftwood exhibited inhibitory activity against CD45 [36], an essential transmembrane protein tyrosine phosphatase associated with T- and B-cell antigen receptor signaling. Discovery work involving another marine fungus, *Phaeosphaeria spartinae* (=*Leptosphaeria spartinae*), isolated from the inner tissues of the marine alga, *Ceramium* sp., resulted in the characterization of eight new natural products, including spartinoxide and spartinol C, demonstrating inhibition of the enzyme human leukocyte elastase; a disease target associated with pulmonary emphysema, rheumatoid arthritis, and cystic fibrosis [37,38]. These examples emphasize that marine fungi *s.s.* can be isolated from a range of different substrata and can produce unique and previously undiscovered natural products. The following text will provide information regarding several methods that are used by mycologists (and some natural product chemists) to isolate truly unique isolates of marine fungi *s.s.*

## 4. Isolation Techniques

### 4.1. Sample Collection

To obtain marine fungi, marine environments with limited human disturbance are preferable collection sites as they support a greater diversity and denser distribution of fungal species [39]. When obtaining samples, aged materials submerged in seawater with periods of exposure in the air are preferable, e.g., as evidenced by the presence of other marine decay organisms, such as barnacles [9]. For wood and macroalgae, samples with a roughened and softened surface may indicate the presence of fungal growth. On rocky shores, wood trapped between crevices of rocks below the tide line support good fungal growth. For sandy beaches, wood/macroalgae buried deep in the sand or the sand grains occurring near these organic substrata are ideal. In mangrove/salt marsh environments, intertidal substrates (i.e., decaying plant parts either attached to the standing plants or those detached and exposed on the sediment floor during low tide) are often teeming with fruiting bodies of marine fungi. Different stages of decay support different fungal species; therefore, an effort should also be made to collect samples from multiple decay stages [40]. Upon collection, samples should immediately be placed in sterile sealed plastic bags containing paper towel wetted with sterile seawater to avoid desiccation [41]. When collected by SCUBA (self-contained underwater breathing apparatus) diving, samples should be placed in sterile sealable plastic tubes or plastic bags while underwater to minimize handling at the surface (to reduce likelihood of exposure to airborne spores). Samples can then be placed on ice for transport to the laboratory for microscopic examination and culturing. It should be noted that slow drying of samples should be avoided prior to processing as the drying process can cause discharge of fungal ascospores [40]. After returning to the lab, if surface fouling organisms are present on samples, they can be scraped off to prevent bacterial growth and decay. Samples should be washed thoroughly in sterile water to remove thick sediment layers and other potentially contaminating debris. Following rinsing, samples should be drained for approximately one hour to remove any excess water [40]. Samples can then be directly examined under a dissecting microscope or placed in an incubation chamber and examined weekly for the presence of fungal reproductive structures, with weekly spraying with sterile seawater to prevent sample desiccation [42]. 

**Figure 4:**
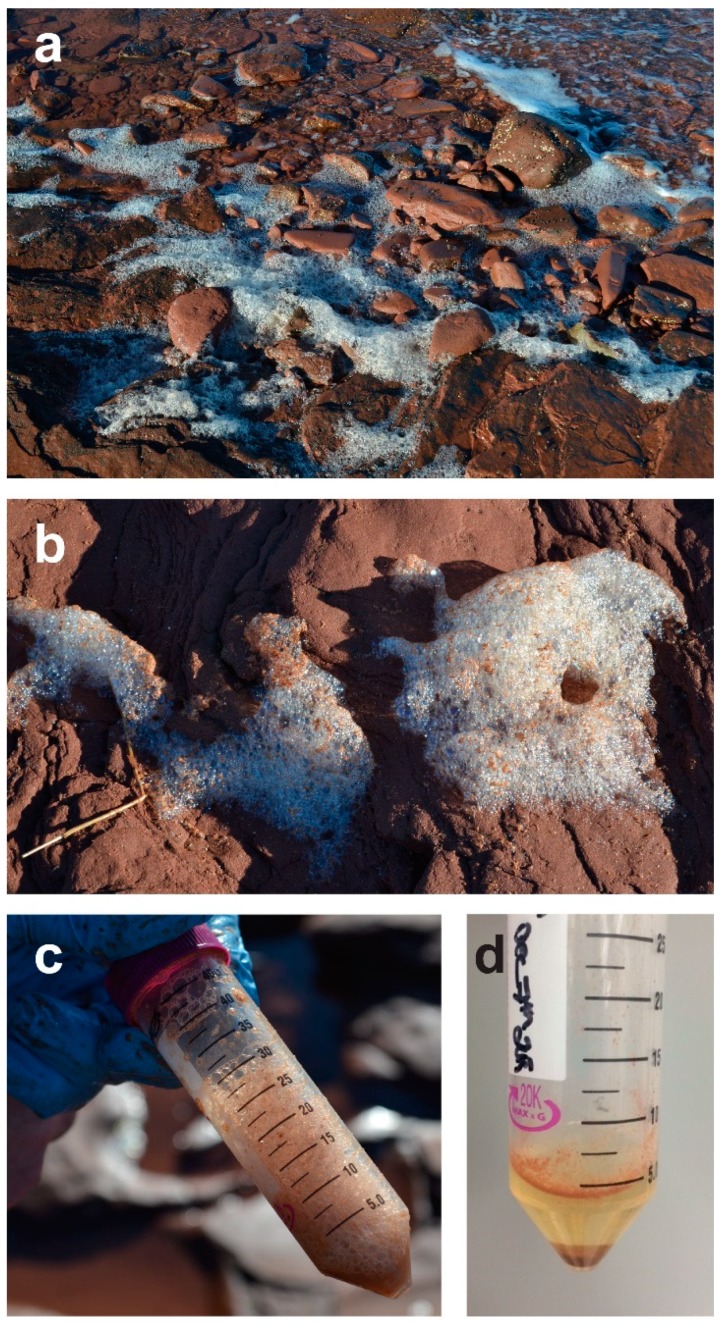
Seafoam as a source of inoculum: (**a**,**b**) Seafoam accumulating onshore during low tide; (**c**) collection of seafoam into sterile centrifuge tubes (care should be taken not to collect any additional sediment or water); (**d**) upon return to lab, a double strength antibiotic in sterile seawater should be added and the seafoam allowed to settle for several hours at 4 °C. Particles/spores can then be concentrated by centrifuging for dilution plating. Note: Storage of samples for longer than a day prior to processing is not recommended, as marine yeasts will continue to grow in the solution, even at refrigeration temperatures and will hamper the isolation of axenic cultures of filamentous fungi.

Sea foam has been historically used to isolate the highly distinctive appendaged conidia of marine arenicolous (sand-dwelling) ascomycetes [9,43,44,45]. During sea foam formation, a large number of fungal propagules become occluded within the air bubbles formed as the waves break upon the shoreline and the bubbles percolate through the shoreline sand [45]. A caveat of using sea foam as an isolation substrate is that fungal propagules present in sea foam are not limited to marine fungi in particular; rather, conidia of terrestrial species (saprophytic and plant pathogenic genera) are often blown in from the shoreline and cosmopolitan generalist fungi naturally growing in the intertidal zone are also frequently isolated [11,43]. Sea foam can be collected by passing through a fine mesh sieve (holes approx. 2mm in diam.) using sterile sea water and collected in a sterile container or collected directly into sterile plastic bottles or centrifuge tubes (Figure 4) [9,11,44]. If the foam has collapsed and dried (such as deposition at high tide), it can be carefully scraped off and resuspended in sterile seawater [9]. The addition of an equivalent portion of a double strength preparation of antibiotic solution (in sterile seawater) prior to direct plating or dilution to extinction culturing is advantageous to limit the growth of bacteria [11]. 

### 4.2. Direct Plating vs. Particle Filtration and Dilution to Extinction Plating

Direct plating techniques offer a means to capture marine fungi that are embedded in their respective substrata and may not be apparent upon visual inspection in the lab. This method is extremely useful for large substrata, such as submerged wood samples, prop roots of mangrove plants, algae, and the stems/roots of salt marsh plants. Fragments (approximately 0.5 cm^2^) can be aseptically cut and placed into petri dishes containing sterile seawater and antibiotics for two to three hours. These fragments can then be subsequently sliced further (to a size of approximately 3 mm^2^) and transferred to agar plates overlaid with sterile seawater and antibiotics for incubation [41]. 

**Figure 5:**
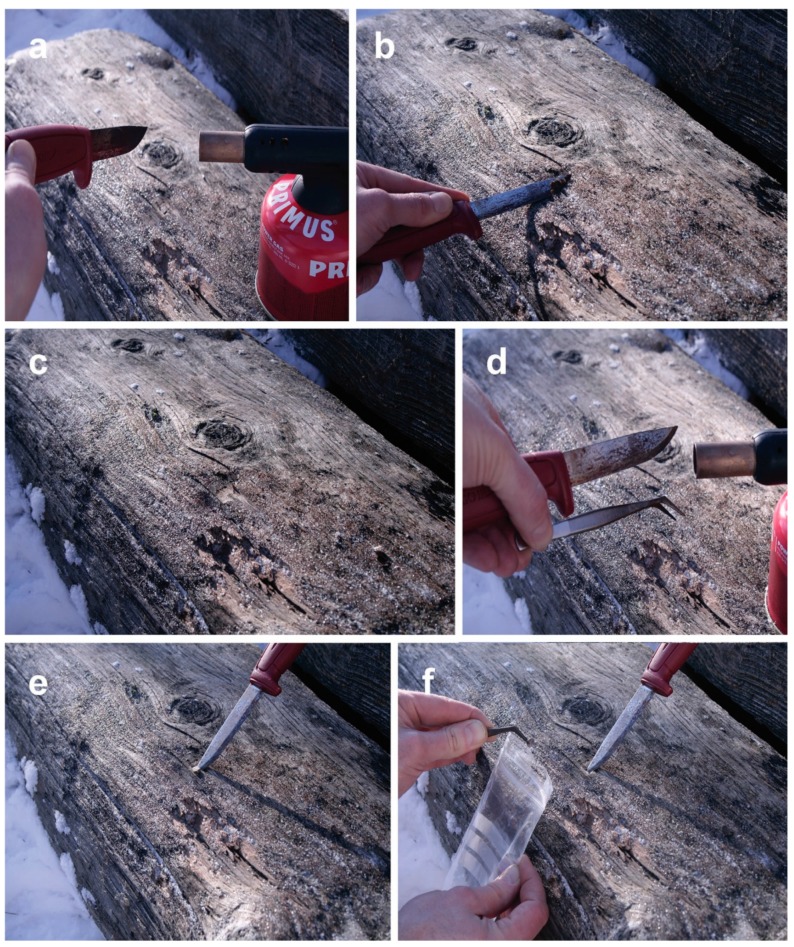
Sampling of marine fungi from wooden logs: (**a**) Flame-sterilization of the sampling knife sprayed with EtOH using a gas burner; (**b**) cutting off a thin slice of surface wood to exclude surface contaminants (using a sharp knife, take as thin a slice as possible to avoid excluding marine fungi inhabiting the upper cell layers below the wood surface); (**c**) a sterile spot on the wooden log prepared for sampling; (**d**) surface sterilization of the knife and forceps with EtOH and the gas burner; (**e**) cutting of a small piece of wood from the wood; (**f**) picking up the wooden piece with forceps and placing it into a sterile plastic bag that is sealed airtight.

Alternatively, samples can be collected in the field (which is ideal when sampling from a substrate that is large and not easily transported to the lab, such as large driftwood samples or logs [46]). In the field, thin slices of wood are cut-off from the surface wood using EtOH and flame sterilized knife and forceps (Figure 5). From the spot where the surface wood is removed, a wooden cube is cut-off and transferred with forceps into sterile plastic bags that are sealed airtight. The sample is kept cool, transported to the laboratory, and plated on agar plates. The plates are monitored over a period of up to several months to allow the appearance of the slowest growing fungi. Mycelia growing out of the wooden cubes are transferred to fresh agar plates and sub-cultured until axenic cultures are obtained. The incubation conditions should mimic the natural conditions as much as possible to select for marine fungi *s.s*. This method has been shown to result in an approximately half of pure culture isolates being marine fungi, including a significant portion of *Sensu stricto* species [46].

**Figure 6:**
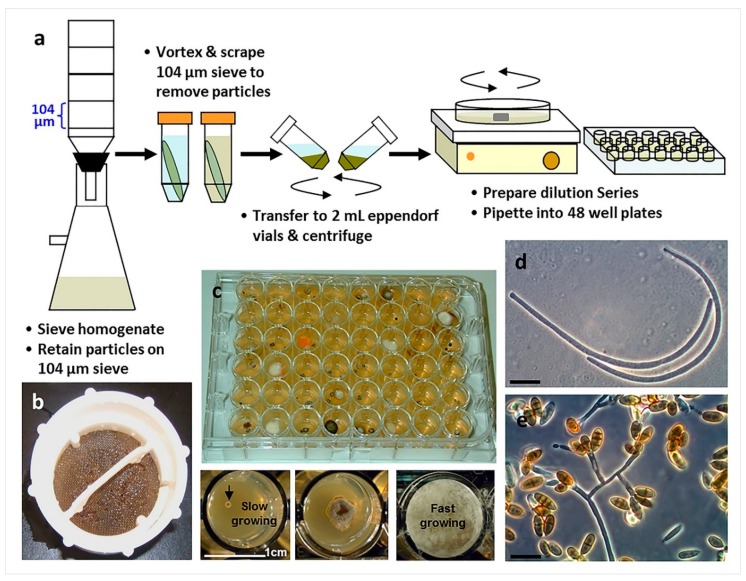
The use of particle filtration and dilution culturing in 48-well plates for the isolation of marine fungi (Overy et al. 2014 [2]): (**a**) Schematic of the particle filtration and dilution plating workflow; (**b**) algal particles that were retained on a filter sieve after homogenization and filtration; (**c**) plating into 48-well plates allows for the separation of fast and slower growing isolates, increasing the isolation frequency of slower growing fungi; (**d**) conidia from a strain of marine fungus, *Anguillospora marina* (telemorph = *Lindra obtusa*), isolated from algal tissue particles (scale bar = 20 µm); (**e**) conidia and conidiophores of a strain of the marine fungus, *Paradendryphiella arenaria*, isolated from algal tissue particles (scale bar = 20 µm).

The combination of substrata particle filtration and dilution-to extinction particle plating is an improvement upon more traditional isolation approaches and has been proven as being effective in obtaining marine fungi and thus merits more wide-spread adoption (Figure 6) [2,47]. Substrata deconstructed by homogenization are filtered and washed through a series of meshes of decreasing pore sizes and particles of a given size are harvested and dispensed at high-dilution rates into 48- or 96-well plates [48,49,50]. This technique increases the rate of capture of fungi originating from vegetative fragments embedded in the substrata; more importantly, partitioning into 48- or 96- well plates allows for slower growing fungi (such as obligate marine fungi) actively growing within substrata particles to be separated and isolated from faster growing taxa. The particle filtration/dilution to extinction culturing approach is ideal for working with marine sediments. Dilution culturing into 48-well plates is a proven method for the successful isolation of marine fungi *s.s.* from seafoam suspensions [11].

### 4.3. Damp Chambers

**Figure 7:**
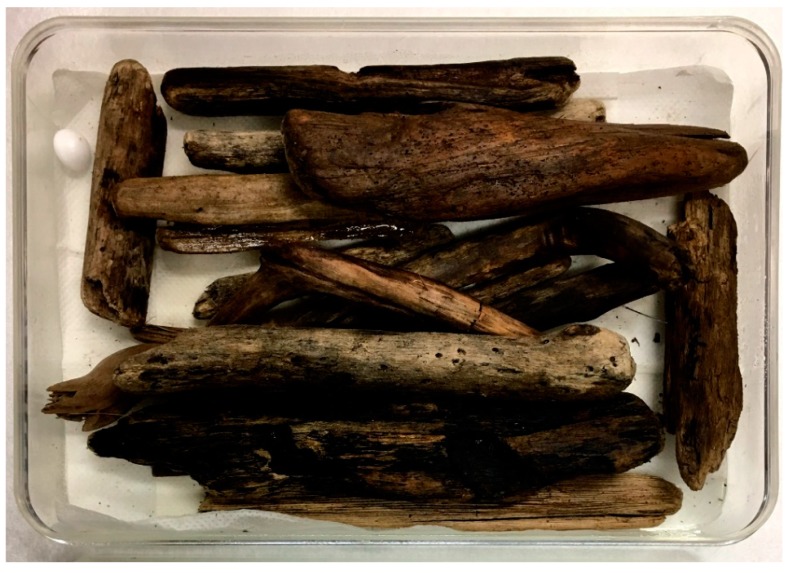
Example of a driftwood damp chamber; note the use of a camphor ball for the prevention of mites.

The incubation of samples in damp chambers provide a moist (high water activity) environment to encourage fungal growth and the formation of sexual fruiting structures and conidiophores in a laboratory setting. Damp chambers consist of a sterilized plastic container (i.e., a Tupperware box, Petri dish, or plastic bag) lined with a sterile, absorbent material (i.e., filter paper, sand, or vermiculite), which is moistened with sterile seawater (Figure 7). Sand or vermiculite are preferable as they are poor growth substrates for bacteria. To prevent bacterial growth, damp chambers should be kept free of accumulated water (and if filter paper is used, it should be moistened with sterile seawater containing antibiotics) [41]. It is important to note that the damp chambering of environmental samples can lead to problematic infestations from mites and other insects (resulting in cross contamination within and between samples), but can easily be prevented by including a small container of camphor in the damp chamber [40]. Damp chambers should be incubated within 5 °C of the ambient temperature at their collection site and sprayed twice weekly with sterile seawater to prevent desiccation [42]. Recommended incubation periods vary between 6–18 months depending on the sample substratum and its length of decay [51]. Samples should be examined periodically under a dissecting microscope for mycelial outgrowth and the development of fungal fruiting bodies or sporulating conidiophores. For example, wood should be examined frequently for 3 months after collection while leaves must be examined at regular intervals within 2 weeks of collection [41,52]. Due to fungal succession (the sequence of fungi sporulating on the same substrate over time), it is important to examine a specimen frequently to maximize the number of species isolated [53]. 

### 4.4. Baiting Stations 

**Figure 8:**
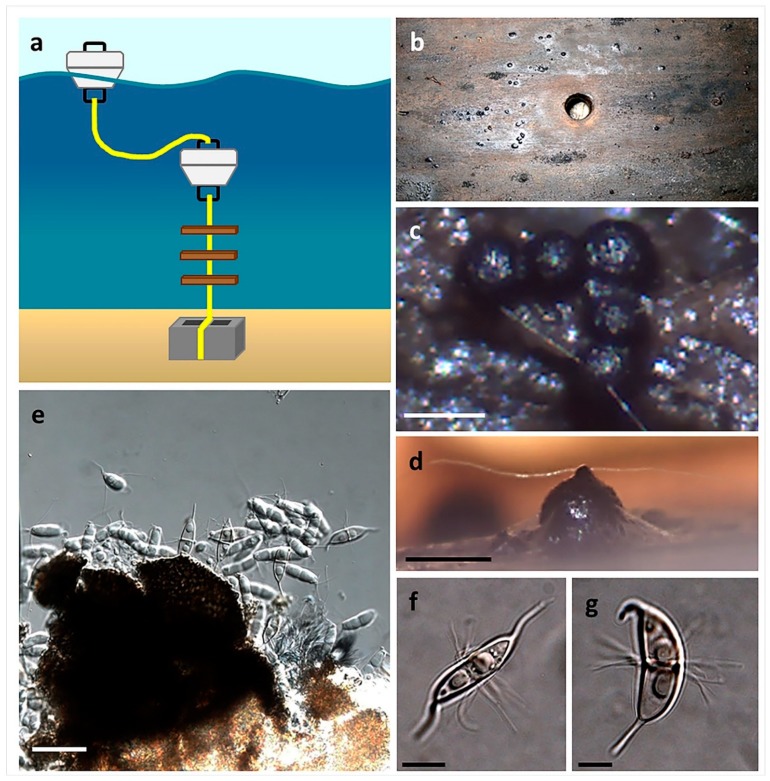
The use of baiting stations for the isolation of marine fungi (Overy et al., 2014 [2]): (**a**) Sterilized wooden boards are suspended in the water column; (**b**) harvested wooden board after 6 months in a damp chamber, ascomata are apparent on the surface; (**c**,**d**) ascomata of *Corollospora maritima* in situ (scale bar = 250 µm); (**e**) squash mount of *C. maritima* ascomata obtained from board (scale bar = 30 µm); (**f**,**g**) ascospores of *C. maritima* with characteristic polar and equatorial appendages (scale bar = 10 µm).

Stations can be created using various baits, such as stems of salt marsh plants or wood (i.e. beech, maple, pine, or balsa wood). Baits should first be sterilized by autoclaving and then strung on a line (i.e., nylon rope or fishing line), a few inches apart, and submerged approximately 0.5 m from the bottom. The use of floats and an appropriate anchor will ensure that the bait lines remain vertical in the water column, do not move their location with the tide, and provide a visual guide to identify their location (Figure 8). The length of submersion time will affect the fungal biodiversity that can be isolated (fungal succession). Over time, baits can also become encrusted with invertebrates, such as barnacles and encrusting tunicates, or become consumed by shipworm, which will hinder the isolation of fungi. One advantage of this technique is that the selection of substrata can be controlled, as hard wood and soft wood can lead to the isolation different species of marine fungi. To harvest, baits are removed from the line and placed into a sterile bag or suitable sterile container for transportation to the lab. In a sterile environment, the bait surface should be scraped with a sterile spatula to remove surface debris and invertebrates (as these organisms will rot and promote significant bacterial growth in the damp chamber) and rinsed several times in sterile seawater. Overnight incubation in plastic bags containing an antibiotic preparation in sterile seawater is effective in reducing the number of viable bacterial cells prior to placing the bait into damp chambers. If using sterile filter paper in the damp chamber, the filter paper should also be saturated with seawater containing antibiotics to prevent the growth of bacteria. Baits should be examined at regular intervals and provided the damp chambers remain humid, be kept for up to a year to monitor for the emergence of different marine fungi [54]. Ideally, damp chambers should be incubated at temperatures proximal to the average temperature range of sea water in which the baiting station was placed [55]. 

## 5. In Situ Culturing 

Direct culturing vs culture independent biodiversity studies predict that only approximately 1% of prokaryotes can be cultured using conventional techniques [56]. Although there are no total estimates for the cultivability of marine fungi, from similar culture vs. culture independent data sets, a low culture recovery (5–7%) has also been observed (Rämä unpublished). Culturing conditions in the laboratory are gross simplifications of natural systems. Many microbes require environmental interactions to grow. Therefore, to access a more bio-diverse range of marine fungi, new and innovative isolation methodologies need to take these factors into consideration. In situ culturing techniques attempt to address these issues and, for bacteriology, have resulted in the successful isolation of previously undescribed organisms [57,58]. Here, suspensions of environmental samples are held between semipermeable membranes and placed back into their native environment. The semipermeable membranes permit the diffusion of growth factors to encourage microbial growth while retaining/preventing the movement or exchange of microbial cells to the environment. It has been shown that microbial cells maintained in in situ culturing devices can, to a significant extent, be domesticated and subsequently subcultured in vitro [57]. There are several existing applications for isolating and cultivating previously uncultivated microorganisms, such as diffusion chambers, its high-throughput version, the Ichip, and the commercially available MicroDish Biochamber [57]. 

**Figure 9:**
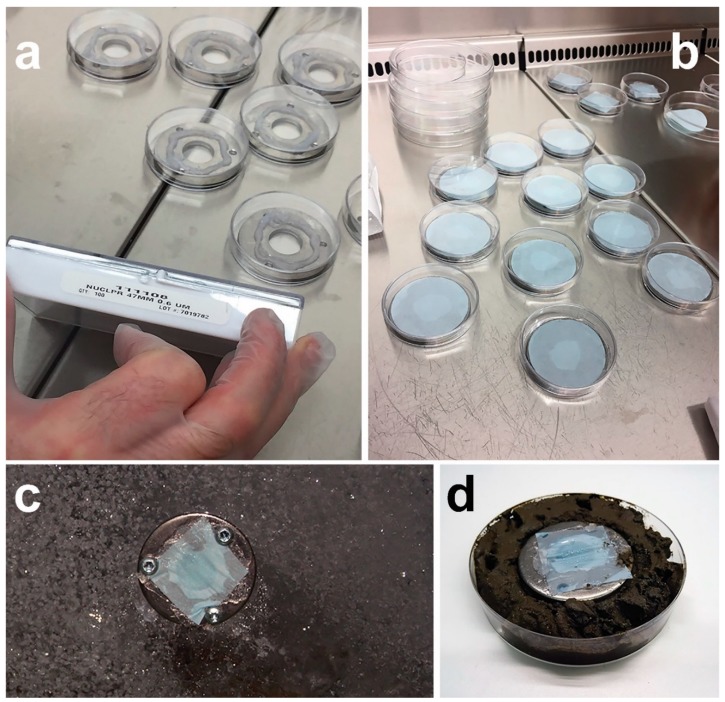
Microbial traps for isolating fungi constructed from stainless steel washer (e.g., 50 × 3 mm in dimensions and with a through whole of 17 mm in diameter) and polycarbonate membranes (e.g., Whatman™ Nuclepore™): (**a**) Steel washers with three drill holes (for securing to solid surfaces, such as driftwood or sea ice), each ready for the construction of a microbial trap under laminar flow; (**b**) using an appropriate adhesive (e.g., aquarium silicon), bottom membranes of the traps with a sufficient pore size to allow fungal filaments to grow into the trap are glued in place (next steps (not pictured) include filling the inside of the washer with agar and, after cooling down of the agar, gluing the bottom membrane of the trap in place); (**c**) using screws, the microbial trap is secured in situ on sea-ice (the figure displays the top of the trap with a small-pored membrane that does not allow movement of the cells from surrounding air into the trap containing agar medium); (**d**) trapping deep-sea sediment fungi using in vitro incubation on sediment sample (again, the top membrane does not allow contaminants to enter the agar medium inside the trap - for a comprehensive description of the construction, see Epstein et al. (2010) [60]).

Whereas the previous applications are based on inoculating cells inside each device that will then be taken out to culture in situ, microbial traps operate according to another principle [59]. An empty device, with growth medium sandwiched between membranes allowing movement of growth factors and/or cells, is taken out to a desired environment. During incubation, filamentous organisms grow into the device through the membrane pores and can later be domesticated in pure cultures in vitro. Using a membrane with a pore size of 0.4–0.6 μm will select for fungi and other filamentous organisms [60]. The selection of a small enough pore size blocks all cell movement and can be used to protect the growth medium inside the device against, e.g., airborne contaminants when trapping sediment fungi. Microbial traps are not available commercially, but they are simple in structure and can be easily built (Figure 9). The construction should take place in a sterile environment to avoid getting contaminants inside the trap. 

## 6. Isolation and Examination of Samples 

Fruiting bodies of marine ascomycetes occur superficially, partially, or fully immersed on/in their respective substrates and when viewed under a stereo microscope, can be isolated directly from substrata. The contents of fruiting bodies or conidia/conidiophores can be aseptically transferred using sterilized fine-tipped forceps or a pin to an appropriate culture medium. Fruiting bodies that are completely immersed within a substratum can be exposed using a sterile razor blade or scalpel. Once exposed, the fruiting body centrum can be transferred to an isolation medium or slide for microscopic examination. For single-spore isolations, centrum material should be first aseptically dispersed in drops of sterile seawater on a sterile glass microscope slide. This spore suspension is then transferred to an appropriate culture medium (i.e., seawater or cornmeal agar containing antibiotics) and monitored daily for the germination of spores using a stereomicroscope. Once observed, individual germinated spores can be transferred by a flame sterilized needle onto a new culture medium and repeatedly sub-cultured to produce axenic cultures [40]. The best time to subculture is while colonies are distinct and autonomous, from areas of newest growth. Mycelial colonies from single spore isolations are preferred to ensure reproducibility in liquid fermentation or solid substrate culturing when screening natural products. For asexual marine fungi with conidia observed from conidiophores appearing on the surface of substrates, individual spores can be picked and inoculated directly onto cornmeal agar plates with antibiotics; alternatively, the single spore isolation method described above can be applied. The few described marine Basidiomycota generally produce reduced size basidiomata, which can be crushed to release spores for identification and isolated as described above.

Various media formulations can be employed for the isolation of marine fungi *s.s.*; however, the choice of isolation medium will have a direct impact on the diversity of fungi obtained [61]. Media that are rich in simple sugars may restrict the growth and sporulation of lignicolous fungi; rather, such rich media often promote the growth of more generalist, cosmopolitan genera, such as *Pencillium* and *Aspergillus*. Lignicolous marine fungi prefer a minimal medium, such as 0.1% glucose, 0.01% yeast extract, 1.8% agar in aged seawater, or adding sterilized birch or balsa wood on top of sea water agar [62]. Seawater or cornmeal seawater agar are commonly used alternatives for the isolation of wood-inhabiting fungi as most species germinate on these media. Agar containing mono- or disaccharides should be avoided if yeast growth is a problem. Specialized carbon sources, such as cornmeal seawater agar or V8 seawater agar, can be used to prevent yeast growth [42,52,63]. Including antibiotics to the isolation media prevents bacterial growth; the addition of cyclohexamide will retard faster, growing generalist, cosmopolitan fungi. Penicillin G (300 mg/L) and streptomycin sulfate (500 mg/L) are effective when used together, added to cooled agar immediately before pouring plates; whereas chloramphenicol can be added to the medium prior to autoclaving. Ideally, inoculated agar plates should be incubated at temperatures close to that of the collection site until sporulation is observed [41]. Once stable cultures are obtained, they can then be transferred to more nutrient rich or specialized media designed for natural product production.

Microscopic examination is an essential step in identifying or confirming the taxonomy of the isolated fungus and is strongly encouraged at the point of initial inoculation, as many fungi produce sterile colonies in culture. Fruiting bodies and conidiophores are placed on a glass microscope slide in a drop of appropriate mounting solution (i.e., lactic acid, lactophenol, cotton blue, sterile sea water, etc.) and covered with a glass cover slip for viewing under a compound microscope. In the case of fruiting structures, such as pycnidia or ascomata, gently pressing on the cover slip will cause the fruiting body to rupture and release its spores, creating a ‘squash-mount’ [9]. As well, centrum contents can be removed directly to a mounting medium for viewing under the microscope. Compound microscopes with a magnification of at least 400 × (ideally 600 × 1000 ×) are best suited for observation of micromorphology as many spores (ie., conidia, ascospores, basidiospores, zygospores, etc.) are less than 20 µm in diameter or length, and information, such as ornamentation, are critical identification characteristics and cannot be resolved at lower magnifications. When using transmitted light microscopy, the use of stains, such as lactophenol cotton blue, aqueous nigrosine, and India ink, can improve contrast. Phase contrast and differential interference contrast microscopy are also useful optical techniques for increasing contrast when viewing specimens. Ascospore appendages and sheaths are often diagnostic for particular species and will only unwind after a period of submersion in water [41]. Dichotomous and pictorial keys based on these fungal reproductive structures are useful identification resources [9,64,65,66]. Semi-permanent microscope slide mounts can be made following the method of Volkmann-Kohlmeyer and Kohlmeyer [67]. 

In addition to micromorphology, identification by DNA sequencing is encouraged. The internal transcribed spacer region of ribosomal DNA is the acknowledged species-level DNA barcode region for fungi and should be sequenced and compared with the reference DNA database, NCBI GenBank, using the BLAST tool [68]. For certain groups of fungi, additional genes may be informative for species-level identifications, e.g., β-tubulin or elongation factor 1-α; for marine yeasts, the 28S ribosomal large subunit region; and for basal fungal lineages, the 18S ribosomal subunit region [69]. Obtained DNA sequences should be deposited in GenBank and associated with vouchered specimen material (dried specimen and/or preserved cultures), as our fungal identifications are only as precise as our reference databases [46]. 

## 7. Preservation

Cryopreservation is preferred for long-term storage of fungal cultures. Agar discs cut from living cultures are placed into sterilized cryoplastic microtubes containing 1 mL of sterile 10% *w*/*v* glycerol as a cryoprotectant [62]. Four to five agar discs should be transferred in a laminar flow cabinet from the growing margin of a fungal culture using a sterilized transfer stick or needle. Microtubes should be stored at 15 °C for 15 min before freezing them at a rate of 1 cm per minute from 15 to −40 °C and 2 cm from −40 to −80 °C. Tubes can then be moved to a storage vessel and preserved in liquid nitrogen at −180 °C. Alternative cryoprotectants include 10% dimethylsulfoxide (DMSO) or 10% glycerol 5% trehalose, which vary in effectiveness depending on the fungal species. For shorter-term storage, agar discs obtained from cultures as above can be stored in sterile distilled water at 4 °C with annual replacement.

Subcultures can also be maintained for storage on agar slants. Slants are made in test tubes with 0.1% agar overlaid with sterile seawater or mineral oil [40,62]. Spores or small colonies are transferred to the surface of the slant using aseptic techniques. The test tube is then capped and stored at 15–20 °C for up to 8 weeks [40]. Alternatively, subcultures can be transferred from agar plates onto potato dextrose or corn meal agar test tube slants and stored at 4 °C permanently (with annual replacement). Cultures can also be preserved using lyophilization (freeze-drying). This method of preservation, however, is only suitable for fungi that sporulate abundantly. As a result, it is not ideal for most marine fungi [62]. To avoid loss of activity through continuous subculturing, multiple first-generation cryostocks should be prepared.

## 8. Concluding Remarks 

It can be argued that “marine-derived” fungi are a proven source of new natural products; this is not in dispute as over a thousand new compounds or new derivatives of known molecules have been discovered over the past few decades. However, recent evidence has demonstrated that the new chemistry is a result of saline stress, where terrestrial counterparts of the same species produce the same chemistry as discovered from marine-derived strains when grown on media containing seawater [70]. Simply mining the terrestrial isolates of osmotolerant species on media containing seawater will achieve the same ends. However, in such a scenario, marine fungi (*Sensu stricto*) will remain an underexplored resource. To address this, attention currently focused on marine derived fungi must shift focus to the isolation of marine fungi (*Sensu stricto*). In addition, new culturing techniques should be adopted and developed for accessing a greater diversity of marine fungi that has been shown to exist using environmental sequencing. 

Assessment of whether marine fungi have evolved to produce secondary metabolites that are distinct from their terrestrial relatives is awaiting completion of genomic sequences. These data will lead to hypotheses about how isolation in the marine habitat may have influenced the evolution of secondary metabolite gene clusters in terms of divergence from ancestral pathways or loss or expansion of pathways for individual structural classes. The principal ascomycete lineages of marine fungi derive primarily from the Sordariomycetidae, Dothideomycetidae, and the Eurotiomycetidae [71,72,73,74,75]. Sequencing of several hundred fungal genomes of representative terrestrial species revealed that the main subclasses of these subphyla often have very rich secondary metabolomes, therefore, it is logical to assume that the marine lineages derived from within these ascomycete subclasses would also have an active and complex secondary metabolism. The few reports of chemistry from acknowledged marine fungi suggest that they will be an excellent source of new chemical entities, often associated with a variety of different biological activities [2]. 

There are several examples in the literature where collaborations between marine mycologists and natural product chemists have resulted in new chemistry discovered from marine fungi [27,32,76,77,78,79]. Hence, we encourage chemists to collaborate with marine mycologists, many of which have personal collections of marine fungi that can be provided for chemical investigation or to acquire marine fungi from recognized culture collections. Through active cooperation and adoption of classical isolation methodologies with modern adaptations, the field of natural products discovery from marine fungi (*Sensu stricto*) will facilitate a deeper exploration of this truly unique and underexplored group of fungi. 

## Figures and Tables

**Table 1:** Summary of isolation techniques of marine fungi *Sensu stricto* presented.

Technique/Substrate	Substrate	References	Notes
Isolation from sea foam	Seafoam	9,11,43–45	Especially suitable for arenicolous species, can yield generalist fungi
Direct plating	Any	41,46	Important to employ multiple media to ensure growth of targeted fungus
Particle filtration and dilution to extinction plating	Any	2,47–50	Especially suited for the isolation of slower growing, unique fungi
Damp chambers	Driftwood, various of macroorganisms	40–42,51–53	Important to employ preventative measures to avoid mite contamination
Baiting stations	Wood	54–55	Encrusting invertebrates can be problematic
In situ culturing	Any	56–60	Novel approach, has the potential to isolate truly unique species

## References

[1] Hover B.M., Kim S.-H., Katz M., Charlop-Powers Z., Owen J.G., Ternei M.A., Maniko J., Estrela A.B., Molina H., Park S. (2018). Culture-independent discovery of the malacidins as calcium-dependent antibiotics with activity against multidrug-resistant Gram-positive pathogen. Nat. Microbiol..

[2] Overy D.P., Bayman P., Kerr R.G., Bills G.F. (2014). An assessment of natural product discovery from marine (*sensu strictu*) and marine-derived fungi. Mycology.

[3] Bugni T.S., Ireland C.M. (2004). Marine-derived fungi: A chemically and biologically diverse group of microorganisms. Nat. Prod. Rep..

[4] Rateb M.E., Ebel R. (2011). Secondary metabolites of fungi from marine habitats. Nat. Prod. Rep..

[5] Tasdemir D. (2017). Marine fungi in the spotlight: Opportunities and challenges for marine fungal natural product discovery and biotechnology. Fungal Biol. Biotechnol..

[6] Karwehl S., Stadler M., Stadler M., Dersch P. (2016). Exploitation of fungal biodiversity for discovery of novel antibiotics. How to Overcome the Antibiotic Crisis.

[7] Jones E.B.G. (2011). Fifty years of marine mycology. Fungal Divers..

[8] Zettler E.R., Mincer T.J., Amaral-Zettler L.A. (2013). Life in the “Plastisphere”: Microbial communities on plastic marine debris. Environ. Sci. Technol..

[9] Kohlmeyer J., Kohlmeyer E. (1979). Marine Mycology: The Higher Fungi.

[10] Jones E.B.G. (1995). Ultrastructure and taxonomy of the aquatic ascomycetous order *Halosphaeriales*. Can. J. Bot..

[11] Overy D.P., Berrue F., Correa H., Hanif N., Hay K., Lanteigne M., Mquilian K., Duffy S., Boland P., Jagannathan R. (2014). Sea foam as a source of fungal inoculum for the isolation of biologically active natural products. Mycology.

[12] Zuccaro A., Mitchell J., Deighton J., White J., Oudemans P. (2005). Fungal communities of seaweeds. The fungal Community: Its Organization and Role in the Ecosystem.

[13] Gessner R.V., Goos R. (1973). Fungi from *Spartina alterniflora* in sRhode Island. Mycologia.

[14] Newell S.Y., Porter D., Lingle W.L. (1996). Lignocellulolysis by ascomycetes (fungi) of a saltmarsh grass (smooth cordgrass). Microsc. Res. Tech..

[15] Kohlmeyer J., Volkmann-Kohlmeyer B. (2001). The biodiversity of fungi on *Juncus roemerianus*. Mycol. Res..

[16] Walker A.K., Campbell J. (2010). Marine fungal diversity: A comparison of natural and created salt marshes of the north-central Gulf of Mexico. Mycologia.

[17] Elmer W.H., Marra R.E. (2011). New species of *Fusarium* associated with dieback of *Spartina alterniflora* in Atlantic salt marshes. Mycologia.

[18] Zhang T., Wang N.-F., Zhang Y.-Q., Liu H.-Y., Yu L.-Y. (2016). Diversity and distribution of aquatic fungal communities in the Ny-Ålesund region, Svalbard (high arctic). Microb. Ecol..

[19] Hassett B.T., Ducluzeau A.-L.L., Collins R.E., Gradinger R. (2017). Spatial distribution of aquatic marine fungi across the western Arctic and sub-arctic. Environ. Microbiol..

[20] Hassett B.T., Gradinger R. (2016). Chytrids dominate arctic marine fungal communities. Environ. Microbiol..

[21] Loque C.P., Medeiros A.O., Pellizzari F.M., Oliveira E.C., Rosa C.A., Rosa L.H. (2010). Fungal community associated with marine macroalgae from Antarctica. Polar Biol..

[22] Rämä T., Hassett B.T., Bubnova E. (2017). Arctic marine fungi from filaments and flagella to operational taxonomic units and beyond. Bot. Mar..

[23] Arvas M., Kivioja T., Mitchell A., Saloheimo M., Ussery D., Penttila M., Oliver S. (2007). Comparison of protein coding gene contents of the fungal phyla Pezizomycotina and Saccharomycotina. BMC Genom..

[24] Lackner G., Misiek M., Braesel J., Hoffmeister D. (2012). Genome mining reveals the evolutionary origin and biosynthetic potential of basidiomycete polyketide synthases. Fungal Genet. Biol..

[25] Ohm R.A., Feau N., Henrissat B., Schoch C.L., Horwitz B.A., Barry K.W., Condon B.J., Copeland A.C., Dhillon B., Glaser F. (2012). Diverse lifestyles and strategies of plant pathogenesis encoded in the genomes of eighteen Dothideomycetes fungi. PLoS Pathog..

[26] Isaka M., Suyarnsestakorn C., Tanticharoen M., Kongsaeree P., Thebtaranonth Y. (2002). Aigialomycins A-E, new resorcylic macrolides from the marine mangrove fungus *Aigialus parvus*. J. Org. Chem..

[27] Isaka M., Yangchum A., Intamas S., Kocharin K., Jones E.G., Kongsaeree P., Prabpai S. (2009). Aigialomycins and related polyketide metabolites from the mangrove fungus *Aigialus parvus* BCC 5311. Tetrahedron.

[28] Vongvilai P., Isaka M., Kittakoop P., Srikitikulchai P., Kongsaeree P., Thebtaranonth Y. (2004). Ketene acetal and spiroacetal constituents of the marine fungus *Aigialus parvus* BCC 5311. J. Nat. Prod..

[29] Kohlmeyer J., Schatz S. (1985). *Aigialus* gen. novo (Ascomycetes) with two marine species from mangroves. Trans. Br. Mycol. Soc..

[30] Tan T.K., Teng C.L., Jones E.B.G. (1995). Substrate type and microbial interactions as factors affecting ascocarp formation by mangrove fungi. Hydrobiologia.

[31] Alias S., Kuthubutheen A., Jones E.G., Alias S.A., Kuthubutheen A.J., Jones E.B.G. (1995). Frequency of occurrence of fungi on wood in Malaysian mangroves. Asia-Pacific Symposium on Mangrove Ecosystems, Developments in Hydrobiology.

[32] Abbanat D., Leighton M., Maiese W., Jones E., Pearce C., Greenstein M. (1998). Cell wall active antifungal compounds produced by the marine fungus *Hypoxylon oceanicum* LL-15G256. J. Antibiot..

[33] Ogita J., Hayashi A., Sato S., Furutani W. (1987). Antibiotic Zopfimarin. Japan Patent.

[34] Kondo M., Takayama T., Furuya K., Okudaira M., Hayashi T., Kinoshita M. (1987). A nuclear magnetic resonance study of Zopfinol isolated from *Zopfiella marina*. Annu. Rep. Sankyo Res. Lab..

[35] Barluenga S., Dakas P.Y., Ferandin Y., Meijer L., Winssinger N. (2006). Modular asymmetric synthesis of aigialomycin D, a kinase-inhibitory scaffold. Angew. Chem. Int. Ed..

[36] Alvi K.A., Casey A., Nair B.G. (1998). Pulchellalactam: A CD45 protein tyrosine phosphatase inhibitor from the marine fungus *Corollospora pulchella*. J. Antibiot..

[37] Elsebai M.F., Kehraus S., Gütschow M., Koenig G.M. (2009). New polyketides from the marine-derived fungus *Phaeosphaeria spartinae*. Nat. Prod. Commun..

[38] Elsebai M.F., Kehraus S., Gütschow M., Koenig G.M. (2010). Spartinoxide, a new enantiomer of A82775C with inhibitory activity toward HLE from the marine-derived fungus *Phaeosphaeria spartinae*. Nat. Prod. Commun..

[39] Hyde K.D., Jones E.B.G., Leaño E., Pointing S.B., Poonyth A.D., Vrijmoed L.L.P. (1998). Role of fungi in marine ecosystems. Biodivers. Conserv..

[40] Bremer G., Hyde K.D., Pointing S.B. (2000). Isolation and culture of thraustochytrids. Marine Mycology—A Practical Approach.

[41] Vrijmoed L.L.P., Hyde K.D., Pointing S.B. (2000). Isolation and culture of higher filamentous fungi. Marine Mycology—A Practical Approach.

[42] Walker A.K. (2012). Marine Fungi of U.S. Gulf of Mexico Barrier Island Beaches: Biodiversity and Sampling Strategy. Ph.D. Thesis.

[43] Koehn R.D. (1982). Fungi isolated from sea foam collected at North Padre island beaches. Southwest. Nat..

[44] Kirk P.W. (1983). Direct enumeration of marine arenicolous fungi. Mycologia.

[45] Hyde K.D., Jones E.G. (1989). Introduction to fungal succession. Bot. J. Linn. Soc..

[46] Rämä T., Nordén J., Davey M.L., Mathiassen G.H., Spatafora J.W., Kauserud H. (2014). Fungi ahoy! Diversity on marine wooden substrata in the high North. Fungal Ecol..

[47] Bills G.F., Christensen M., Powell M., Thorn G., Mueller G., Bills G.F., Foster M. (2004). Saprobic soil fungi. Biodiversity of Fungi, Inventory and Monitoring Methods.

[48] Collado J., Platas G., Paulus B., Bills G.F. (2007). High-throughput culturing of fungi from plant litter by a dilution-to-extinction technique. FEMS Microbiol. Ecol..

[49] Unterseher M., Schnittler M. (2009). Dilution-to-extinction cultivation of leaf-inhabiting endophytic fungi in beech (*Fagus sylvatica* L.)—Different cultivation techniques influence fungal biodiversity assessment. Mycol. Res..

[50] Shrestha P., Szaro T.M., Bruns T.D., Taylor J.W. (2011). Systematic search for cultivatable fungi that best deconstruct cell walls of *Miscanthus* and sugarcane in the field. Appl. Environ. Microbiol..

[51] Jones E.B.G. (2000). Marine fungi: Some factors influencing biodiversity. Fungal Divers..

[52] Pang K.-L., Chow R., Chan C., Vrijmoed L. (2011). Diversity and physiology of marine lignicolous fungi in Arctic waters: A preliminary account. Polar Res..

[53] Hyde K.D., Jones E.B.G. (2002). Introduction to fungal succession. Fungal Divers..

[54] Shearer C.A. (1972). Fungi of the Chesapeake bay and its tributaries. III. The distribution of wood-inhabiting ascomycetes and fungi imperfecti of the Patuxent river. Am. J. Bot..

[55] Lamore B.J., Goos R.D. (1978). Wood-inhabiting fungi of a freshwater stream in Rhode Island. Mycologia.

[56] Rappé M.S., Giovannoni S.J. (2003). The uncultured microbial majority. Annu. Rev. Microbiol..

[57] Nichols D., Cahoon N., Trakhtenberg E.M., Pham L., Mehta A., Belanger A., Kanigan T., Lewis K., Epstein S.S. (2010). Use of Ichip for high-throughput in situ cultivation of “uncultivable” microbial species. Appl. Environ. Microbiol..

[58] Ling L.L., Schneider T., Peoples A.J., Spoering A.L., Engels I., Conlon B.P., Mueller A., Schäberle T.F., Hughes D.E., Epstein S. (2015). A new antibiotic kills pathogens without detectable resistance. Nature.

[59] Gavrish E., Bollmann A., Epstein S., Lewis K. (2008). A trap for in situ cultivation of filamentous actinobacteria. J. Microbiol. Methods.

[60] Epstein S.S., Lewis K., Nichols D., Gavrish E., Baltz R.H., Demain A.L., Davies J.E., Bull A.T., Junker B., Katz L., Lynd L.R., Masurekar P., Reeves C.D., Zhao H. (2010). New approaches to microbial isolation. Manual of Industrial Microbiology and Biotechnology.

[61] Mueller G.M., Bills G.F., Foster M.S. (2004). Biodiversity of Fungi: Inventory and Monitoring Methods.

[62] Kirk P.W. (1969). Isolation and culture of lignicolous marine fungi. Mycologia.

[63] Nakagiri A., Jones E.G., Hyde K.D., Pointing S.B. (2000). Long-term maintenance of cultures. Marine Mycology—A Practical Approach.

[64] Hyde K.D., Sarma V.V., Hyde K.D., Pointing S.B. (2000). Pictorial key to higher marine fungi. Marine Mycology—A Practical Approach.

[65] Jones E.B.G., Sakayaroj J., Suetrong S., Somrithipol S., Pang K.-L. (2009). Classification of marine Ascomycota, anamorphic taxa and Basidiomycota. Fungal Divers..

[66] Kohlmeyer J., Volkmann-Kohlmeyer B. (1991). Illustrated key to the filamentous higher marine fungi. Bot. Mar..

[67] Volkmann-Kohlmeyer B., Kohlmeyer J. (1996). How to prepare truly permanent microscope slides. Mycologist.

[68] Schoch C.L., Seifert K.A., Huhndorf S., Robert V., Spouge J.L., Levesque C.A., Chen W., Consortium F.B. (2012). Nuclear ribosomal internal transcribed spacer (ITS) region as a universal DNA barcode marker for Fungi. Proc. Natl. Acad. Sci. USA.

[69] Fell J.W., Jones E.B.G., Pang K.-L. (2012). Yeasts in marine environments. Marine Fungi and Fungal-like Organisms.

[70] Overy D.P., Correa H., Roullier C., Chi W.-C., Pang K.-L., Rateb M., Ebel R., Shang Z., Capon R., Bills G.F. (2017). Does osmotic stress affect natural product expression in fungi?. Mar. Drugs.

[71] Campbell J., Shearer C.A., Mitchell J.I., Eaton R.A., Hyde K.D., Pointing S.B. (2000). Corollospora revisited: A molecular approach. Marine Mycology—A Practical Approach.

[72] Schoch C.L., Sung G.-H., Volkmann-Kohlmeyer B., Kohlmeyer J., Spatafora J.W. (2007). Marine fungal lineages in the Hypocreomycetidae. Mycol. Res..

[73] Schoch C.L., Sung G.-H., López-Giráldez F., Townsend J.P., Miadlikowska J., Hofstetter V., Robbertse B., Matheny P.B., Kauff F., Wang Z. (2009). The Ascomycota tree of life: A phylum-wide phylogeny clarifies the origin and evolution of fundamental reproductive and ecological traits. Syst. Biol..

[74] Zuccaro A., Schoch C.L., Spatafora J.W., Kohlmeyer J., Draeger S., Mitchell J.I. (2008). Detection and identification of fungi intimately associated with the brown seaweed *Fucus serratus*. Appl. Environ. Microbiol..

[75] Suetrong S., Schoch C.L., Spatafora J.W., Kohlmeyer J., Volkmann-Kohlmeyer B., Sakayaroj J., Phongpaichit S., Tanaka K., Hirayama K., Jones E.B.G. (2009). Molecular systematics of the marine *Dothideomycetes*. Stud. Mycol..

[76] Abraham S., Hoang T., Alam M., Jones E.G. (1994). Chemistry of the cytotoxic principles of the marine fungus *Lignincola laevis*. Pure Appl. Chem..

[77] Lin Y., Wu X., Deng Z., Wang J., Zhou S., Vrijmoed L., Jones E.G. (2002). The metabolites of the mangrove fungus *Verruculina enalia* No. 2606 from a salt lake in the Bahamas. Phytochemistry.

[78] Poch G.K., Gloer J.B. (1989). Obionin A: A new polyketide metabolite form the marine fungus *Leptosphaeria obiones*. Tetrahedron Lett..

[79] Poch G.K., Gloer J.B. (1989). Helicascolides A and B: New lactones from the marine fungus *Helicascus kanaloanus*. J. Nat. Prod..

